# Tracing heavy metals in ‘swine manure - maggot - chicken’ production chain

**DOI:** 10.1038/s41598-017-07317-2

**Published:** 2017-08-21

**Authors:** Wanqiang Wang, Wenjuan Zhang, Xiaoping Wang, Chaoliang Lei, Rui Tang, Feng Zhang, Qizhi Yang, Fen Zhu

**Affiliations:** 1 Hubei International Scientific and Technological Cooperation Base of Waste Conversion by Insects, Huazhong Agricultural University, Wuhan, Hubei Province 430070 China; 2 Hubei Insect Resources Utilization and Sustainable Pest Management Key Laboratory, Huazhong Agricultural University, Wuhan, Hubei Province 430070 China; 3 MoA-CABI Joint Laboratory for Bio-safety, Institute of Plant Protection, Chinese Academy of Agricultural Sciences, 2 West Yuan-ming-yuan Road, Beijing, 100193 China; 4 School of Mechanical Engineering, Jiangsu University, Zhen Jiang, Jiangsu Province 212013 China; 5 State Key Laboratory of Integrated Management of Pest Insects and Rodents, Institute of Zoology, Chinese Academy of Sciences, 1-5 West Beichen Road, Beijing, 100101 China

## Abstract

With the development of large-scale livestock farming, manure pollution has drawn much attention. Conversion by insects is a rapid and cost-effective new method for manure management. Swine manure conversion with maggots (*Musca domestica* larvae) has developed, and the harvested maggots are often used as animal feed. However, the flow of heavy metals from manure to downstream processes cannot be ignored, and therefore, heavy metal content was measured in untreated raw manure, maggot-treated manure, harvested maggots and maggot-eating chickens (chest muscle and liver) to evaluate potential heavy metal risks. The levels of zinc, copper, chromium, selenium, cadmium and lead had significant differences between untreated raw manure and maggot-treated manure. The concentrations of all detected heavy metals, except for cadmium and selenium, in maggots met the limits established by the feed or feed additive standards of many countries. The bioaccumulation factor (BAF) of heavy metals decreased with the increase of the maggot instar, indicating that heavy metals were discharged from the bodies of maggots with the growth of maggots. Also, the contents of overall heavy metals in chickens fed harvested maggots met the standards for food. In conclusion, regarding heavy metals, it is eco-safe to use maggots in manure management.

## Introduction

Manure management has become an important part of environmental protection^1–3^. Livestock production generates approximately 1400 million tons of manure annually in the European Union (EU)^4^ and excess manure produced can be used as a resource. To date, widely employed technologies involve fertilizer treatment, energization, and compressive processes^5^. In many countries, for example, in France^6, 7^, animal manures are mainly used as organic fertilizers for agricultural production because of their abundant nutrient compositions, such as nitrogen (N), phosphorus (P) and potassium (K). However, several substances contained in manure that are harmful to human safety as well as ecosystems (heavy metals, antibiotics and pathogens) limit this development^6, 8–11^. Cang *et al*.^1^ detected high concentrations of heavy metals (Zinc (Zn), copper (Cu), chromium (Cr), cadmium (Cd), lead (Pb), nickel (Ni) and arsenic (As)) in swine manure, and these levels were much higher than those in feeds of Jiangsu Province, China^1^. Researchers also found that heavy metals would enter into the environment from manure, and the levels of Cu, Zn, As, Cd and Cr in manure increased rapidly from 1990 to 2010, especially in pig and poultry manure^12, 13^. In addition, the application of swine manure, which contains high levels of NPK, to agricultural production without treatment leads to eutrophication^14^. It is reported that the land application of manure can cause N leaching, ammonia (NH_3_) volatilization, and nitrous oxide (N_2_O) emission^15–18^. A survey of groundwater NO_3_^−^-N concentrations showed that up to 55% of 394 groundwater samples on the North China Plain exceeded the WHO drinking water standard of 11.3 mg N L^−1^ for NO_3_^−^-N^19^. Although a large portion of manure produced has been applied back to the field as fresh or dried fertilizer, manure remains an important source of pollution. Relatively harmless, improved resource utilization solutions for manure management are needed for the sustainable development of livestock farming.

A rapid, cost-effective method of manure management is conversion by insects. Many insects can reduce the volume of the manure and transform it into high-quality fertilizer^20^. The housefly (*Musca domestica*) is considered a good candidate for use in manure management. A specific weight ratio of neonate maggots is added to fresh swine manure, and after 7 days under ambient conditions, all the late instar maggots (near pupation) are separated from the manure manually by gradually removing the surface manure with a broom because of their negative phototaxis^21^. In a previous study, the total weight of manure reduced 1.12 ± 0.10 kg per day when treated with 1 kg maggots during vermicomposting, and an 80.2% reduction in moisture^22^. Moreover, the microbial activity, observed as basal respiration, in maggot-treated manure decreased by 35%, and the microbial diversity decreased from 2.57 in raw manure to 1.77 on the Shannon index^22^. Zhu *et al*. (2015) also found the total N, P and K content in maggot-treated organic fertilizer was 10.72% (dry weight), which was higher than that in organic fertilizer produced by conventional composting with bulking agents (approximately 8.0%)^21^. The insects can be harvested afterwards and used as animal feed^23–25^. Zhu *et al*. (2015) dried the fresh maggots harvested from manure using microwave equipment and measured the nutrient contents, 55.32 ± 1.09% protein was contained in the dried maggots, which included 1.34 ± 0.02% methionine and 4.15 ± 0.10% lysine^21^, indicating that larval feeding had converted organic waste into a highly valuable protein that can be used as a substitute for fishmeal. This waste treatment technology may contribute to a reduction of the animal protein shortage in the animal feed market, but there are potential risks associated with these harvested insects, including allergens, chemical hazards (heavy metals, toxins, veterinary drugs, hormones and others), and biological hazards (bacteria, viruses, parasites, fungi, prions), which are not yet well understood^5^. The environmental pollutants could be transferred from different substrates to farmed insects and then to farmed animals through the food chain, so risk assessments of the pollutants should be performed before the insects used as feed.

One of the main limiting factors in the use of manure compost is the concentration of heavy metals^26^. Heavy metals are widespread in animal feeds, faeces, and *in vivo*. Liu *et al*.^27^ detected arsenobetaine (AsB), dimethylarsinic acid (DMA^V^) and monomethylarsonic acid (MMA^V^) (30.99, 1.32 and 1.14 μg/kg, respectively) in chicken breasts after the animals were fed with basal diets that had trace concentrations of AsB (0.03–0.1 μg/g), arsenate (AsV; 0.04–0.1 μg/g), and DMA^V^ (0.03–0.04 μg/g)^27^. The effect of a metal element on an organism is related to its properties. Elements such as selenium (Se), Cu, and Zn are essential in trace quantities for the maintenance of cellular processes. However, other metallic elements (Cd and Pb) and metalloid As may have no function within the body or may even be harmful to human health^28^. In order to improve production efficiency in the livestock production, the addition of different heavy metals in the animal feed is a common method. Cu (mostly in the form of copper sulphate) is commonly used as a footbath in milking yards to treat lameness in dairy cattle and as a growth promoter in pig and poultry systems^29^. Zn (mostly in the form of zinc oxide) is added to pig feeds as a “cure-all” for scour. Only 50 mg/kg addition is beneficial for poultry growth, feather and skeletal development as well as reproduction^30^. Meanwhile ingestion of Cd-contaminated substances may affect the function of major organs such as the brain, kidneys, liver, spleen and testes in an *in vivo* murine model^31–33^. Tyrrell *et al*.^34^ treated rats with 0.05% w/v Pb acetate in their drinking water for 8 weeks and found that the rats developed induced fasting hyperglycaemia^34^. Elements that have entered into organisms can also be biotransformed to other compounds, with resulting changes in toxicity. Yang *et al*.^35^ detected N-acetyl-4-hydroxy-m-arsanilic acid (N-AHAA), inorganic arsenite (AsIII), dimethylarsinic acid (DMAV), 3-amino-4-hydroxyphenylarsonic acid (3-AHPAA), arsenate (AsV), monomethylarsonic acid (MMAV), and 3-nitro-4-hydroxyphenylarsonic acid (ROX) in chicken litter even though the feed only contained ROX^35^. The ability of organisms to adapt to heavy metals is limited, biological function will be destroyed if excessive heavy metals are ingested. For security reasons, all countries have developed standards for heavy metals in manure, fertilizer and feed, and products whose heavy metal levels exceed the standards will be banned from use in agricultural production.

To assess the potential risk of heavy metals transformed by maggots in swine manure and the possibility of harvested maggots being made into feed for livestock farming, we converted swine manure using maggots according to a specific weight ratio, and the harvested maggots were then utilized for feeding chickens. In the process, the swine manure, maggots, chickens and chicken faeces were randomly sampled for detection of heavy metals (Zn, Cu, Cr, Cd, Pb, Ni, As and Se). In addition, to compare the ability of different ages of maggots to convert heavy metals, we calculated the bioaccumulation factors (BAF) of heavy metals in different ages of maggots. Oral ingestion is the primary route of contaminant uptake by animals^36^, and the subsequent bioaccumulation refers to both bioconcentration and biomagnification^37^. Thus, the bioaccumulation factor (BAF) is developed by calculating the concentration of a pollutant in an organism divided by its concentration in the organism’s diet^38, 39^.

## Results

### Heavy metal levels in manure

**Figure 1:**
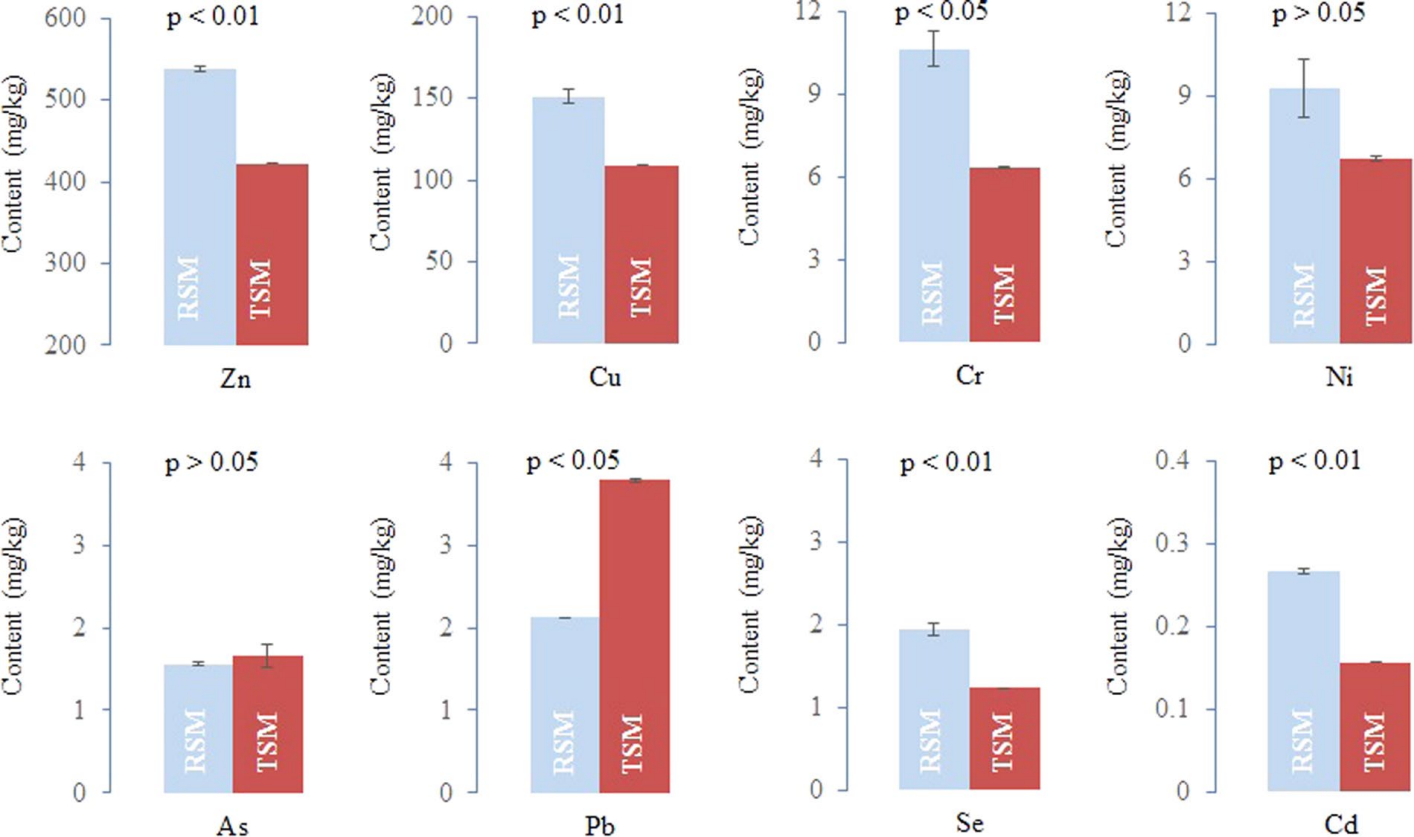
Heavy metal levels in swine manure before and after maggot conversion. RSM means raw swine manure; TSM means maggot-treated swine manure; data are presented as the means ± SEM (n = 3); the p value represents the significant differences; and values were analysed by Student’s t-test.

The results revealed intriguing differences between the heavy metal levels of raw manure and maggot-treated manure (Fig. 1), with remarkably lower levels of Zn, Cu, Cr, Se, and Cd found in the maggot-treated manure compared to the raw manure. The content of Cr in the manure was reduced by 40 percent after maggot conversion, but no significant difference in Ni content was found. The level of As in the manure also did not change before and after maggot conversion. Unexpectedly, lead (Pb) was condensed in the maggot-treated manure.

### Heavy metal transferred to maggots

**Figure 2:**
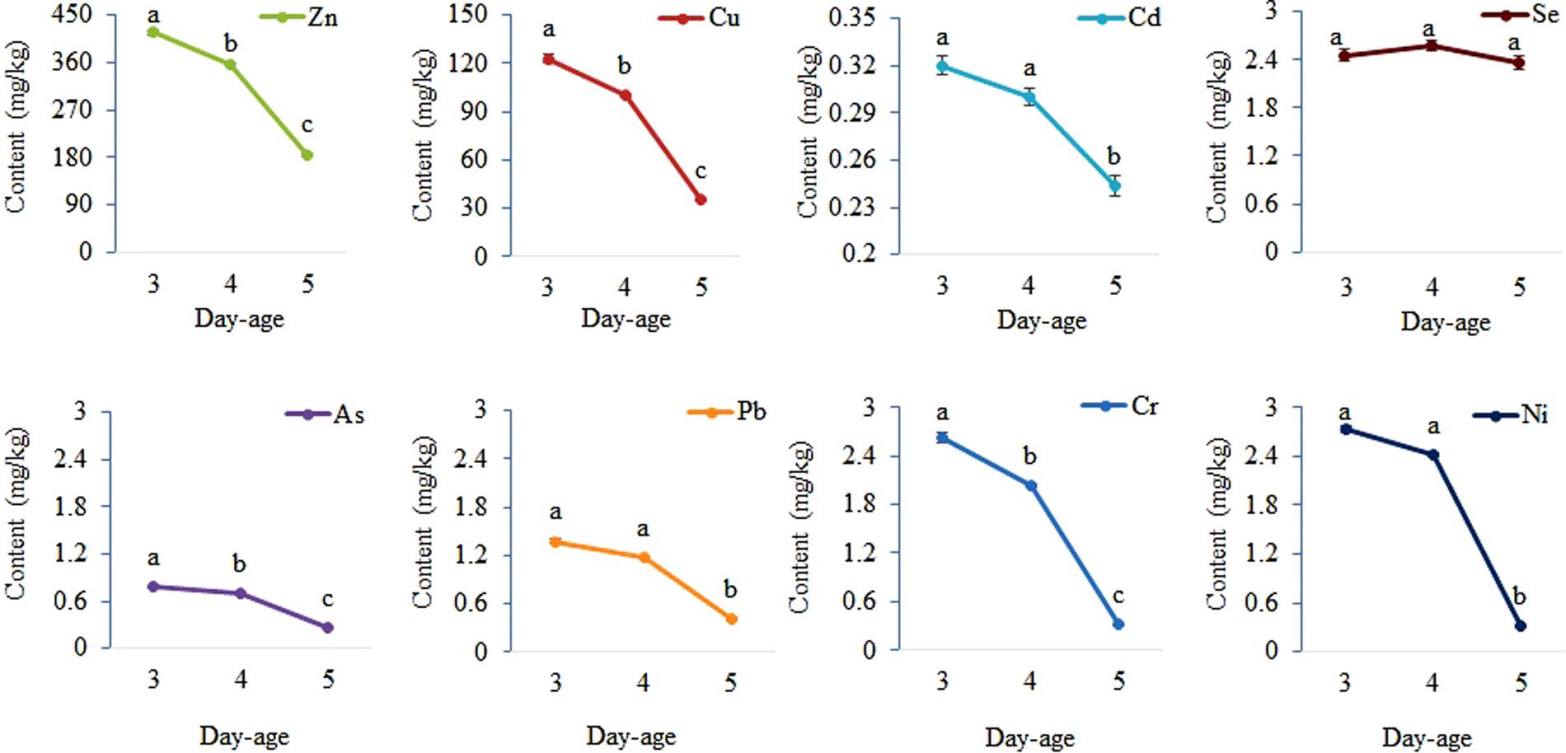
Heavy metal levels in maggots of different ages (days) fed swine manure. The mass represents the average content of heavy metals in maggots of different ages; the different letters on each line indicate the significant differences between treatments at P < 0.05 during different ages in days.

Some heavy metals were found to be present in *M. domestica* larvae (Fig. 2). From 3 to 5 days old, the heavy metal content per unit of body mass decreased as age increased. Except for Se, five-day-old larvae exhibited the lowest heavy metal levels, and larvae of different ages exhibited strong differences in the content of Zn, Cu, As, and Cr. The levels of Cd, Pb, and Ni between 3-day-old and 4-day-old larvae were not strikingly different but were significantly higher than in 5-day-old larvae.

### Bioaccumulation factors (BAF) of maggots

Younger larvae had higher heavy metal BAF (Table 1). The 3-day-old and 4-day-old larvae exhibited high BAF for Cd (1.20 and 1.10, respectively), while the 5-day-old larvae exhibited <1 BAF for Cd. The BAF was very low for Cr (BAF = 0.25–0.03) and Ni (BAF = 0.29–0.03) and remained >1 (1.21–1.32) for Se.

**Table 1:** Heavy metal bioaccumulation factors (BAF) in maggots fed with swine manure.

Heavy metals	Day-age		
	3	4	5
Cr	0.25	0.19	0.03
Cu	0.81	0.66	0.23
Zn	0.77	0.66	0.35
As	0.50	0.45	0.17
Cd	1.20	1.10	0.90
Pb	0.65	0.55	0.19
Ni	0.29	0.26	0.03
Se	1.26	1.32	1.21

### Heavy metals in chicken faeces

**Figure 3:**
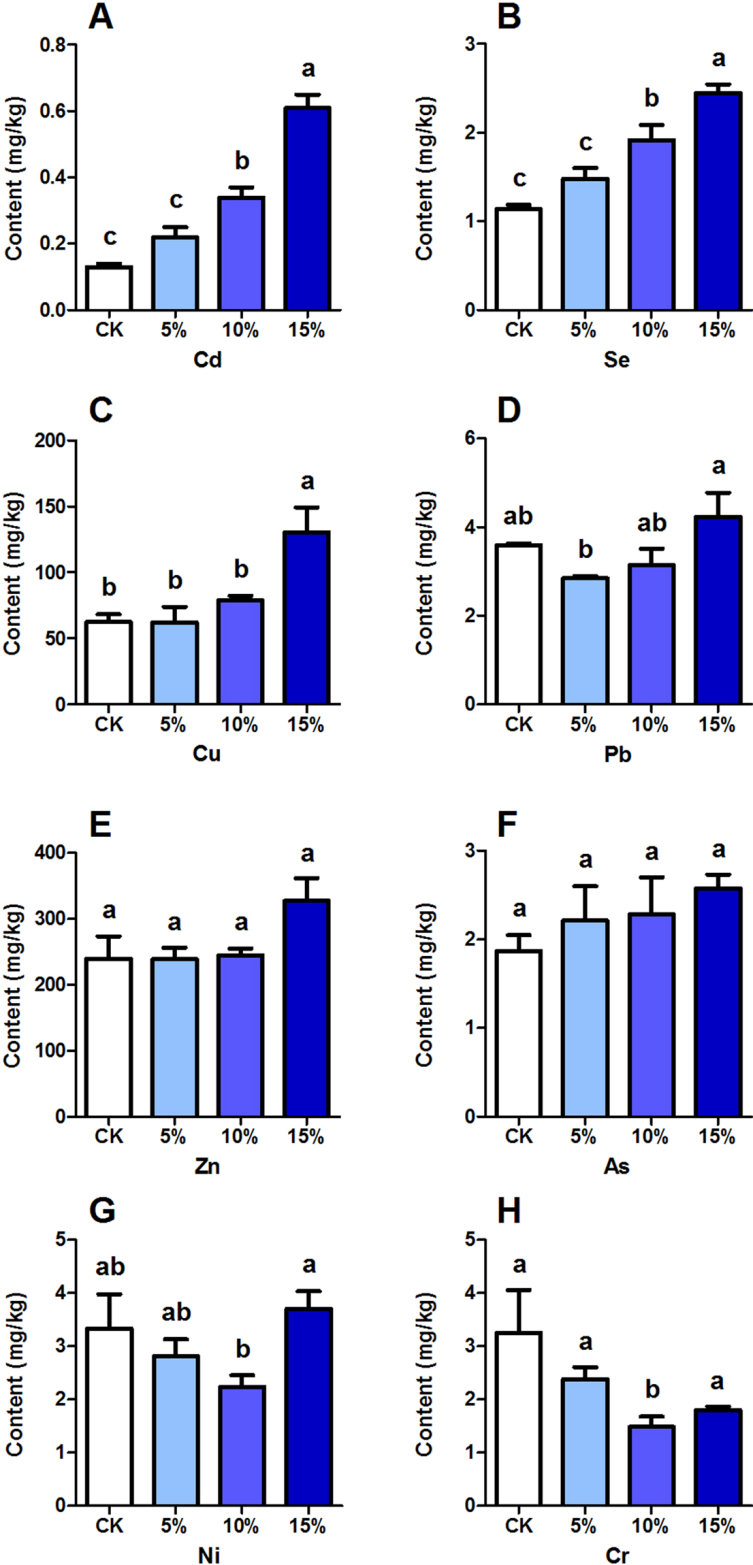
Heavy metal concentrations in the faeces of chickens fed with maggot meal. The x-axis in all plots shows the proportions of maggots added into the feed (0%, 5%, 10% and 15%). CK represents meal feed for chickens without maggot addition. Data are means ± SEM (n = 3). Different letters on each column indicate significant differences between treatments at P < 0.05 during different groups.

The trends of heavy metal levels in the faeces of chickens were not quite consistent with the increased usage of maggots (Fig. 3). Based on the results of our experiment, the levels of Cd, Se, Pb and Cu in chicken faeces increased significantly when more maggots were used for chicken feeding. The levels of elements Zn and As didn’t change and the levels of Ni and Cr even decreased when maggot use increased from 0% to 10% in the chicken feed. However, for the overall elements, the average levels in the 15% treatment groups were higher than those in the 10% treatment groups. Comparing the data with the contents of heavy metals in raw manure (Fig. 1) found that the Zn, Cu, Cr and Ni levels were significantly reduced, and in contrast, higher concentrations of As, Pb, Se, Cd were detected in chicken faeces fed with 15% maggot meal than in raw manure.

### Heavy metal in the meat (chest muscles) and livers of chicken

**Figure 4:**
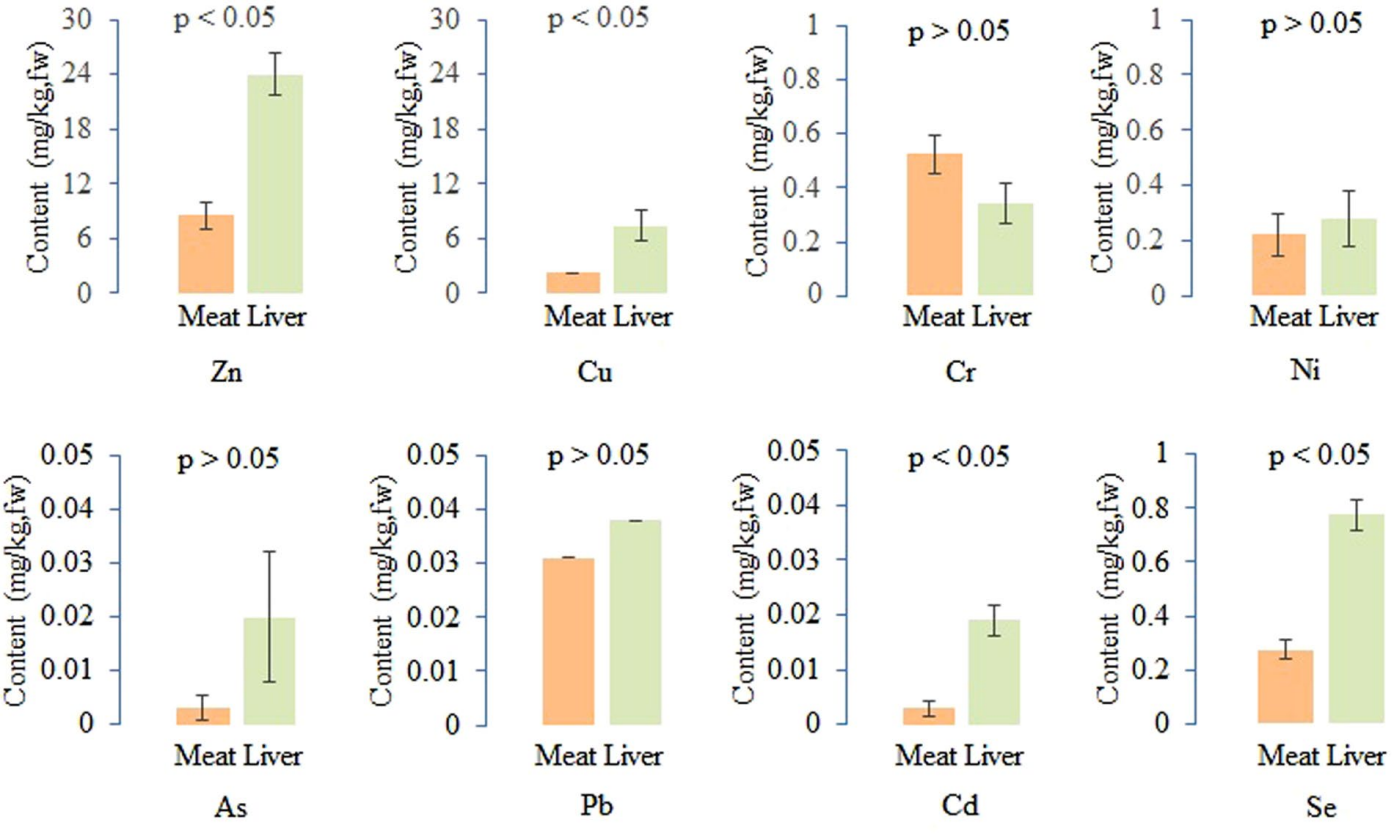
Heavy metal concentrations in the meat (chest muscle) and livers of chickens fed with 15% maggot meal harvested from swine manure. Data are presented as the means ± SEM (n = 3), and the p value represents the significant differences analysed with an *ANOVA* test.

We detected very low heavy metal concentrations in the meat and livers of chickens fed with 15% maggot meal harvested from swine manure (Fig. 4). The levels of Zn, Cu, Se, and Cd were much higher in the liver than in the meat, but there were no significant differences in the contents of Cr, Ni, As, and Pb.

## Discussion

Many heavy metals accumulate in animal manure. In our study, the concentrations of Zn and Cu (538.29 mg/kg and 151.11 mg/kg, respectively) in raw manure were significantly higher than that of Cr, Ni, As, Pb, Se, and Cd (10.64 mg/kg, 9.27 mg/kg, 1.56 mg/kg, 2.12 mg/kg, 1.95 mg/kg, and 0.27 mg/kg, respectively). Similar results were found in research by Sager (2007); the median Cu and Zn contents in swine manure were 282 and 1156 mg/kg, respectively, in Austria, but the contents of Se, As, Cd, Cr, Ni, and Pb were lower with median values of 3.37, 0.88, 0.46, 6.9, 12.5, and 1.9 mg/kg^40^, respectively. After manure was treated with maggots in our experiment, we were pleased to find most concentrations of selected heavy metals (Zn, Cu, Cr, Se, and Cd) decreased, with values of 422.91, 108.85, 6.34, 1.24 and 0.16 mg/kg, respectively. No significant differences were found in As and Ni with values of 1.66 and 6.37 mg/kg, respectively. What was puzzling was that higher level of Pb (3.78 mg/kg) was detected in the maggot-treated manure, indicating that maggots have little ability to transform Pb in manure. Diener *et al*.^38^ reported higher Pb concentrations in the larval exuviae than in the larvae or their feed, indicating that Pb is sequestered in the exoskeleton of the black soldier fly (*Hermetia illucens*)^41^. Vijver *et al*.^42^ also found the Pb concentration in the body of *Tenebrio molitor* was far lower than in their feed^42^. We suspect that only a little Pb was absorbed by the maggots and the excess Pb was quickly returned to the environment through moulting or excretion, so the content of Pb in the manure increased with the decrease of manure quality. The contents of heavy metals (Zn, Cu, Cr, Cd, Pb, Ni and As) in the composting process have been detected in previous work by Han *et al*. (2008), who found that the overall content of heavy metals, except for As, increased in animal manure after 63 days of composting^43^. The concentrations of Cr, Ni, Cu, Zn, Cd, Pb and As were 12.9, 10.9, 465.7, 656.7, 1.09, 38.2 and 21.8 mg/kg, respectively, compared to 8.0, 9.2, 185.2, 194.4, 0.52, 11.1 and 28.0 mg/kg, respectively, in raw manure^43^. Additional results were found in the work of Li *et al*.^44^, where the content of Cu bound to organic matter in swine manure increased from 60% to 75% after transit through the gut of earthworms, whereas Zn decreased from 50% to 25%^44^. Comparing the concentrations of these heavy metals in swine manure after maggot conversion to the standards established by China, Canada (Table 2), and some European countries (Table 3)^45, 46^, all the heavy metal levels met the national standards except those of Cu and Zn (Cu < 100 mg/kg and Zn < 400 mg/kg in Germany). In conclusion, maggots can effectively transform the heavy metals in swine manure, especially Zn, Cu, Cr, Se, and Cd. Although the contents of Ni and As in swine manure could not be reduced with maggot treatment, the transformation efficiency was higher than that of natural compost.

**Table 2:** Heavy metal limits in fertilizer, animal feed and human food (mg/kg). ‘—’ means the standard of relevant heavy metal concentration have not been stipulated. ^a^Source: Standard for microbial organic fertilizers, NY 884–2012, China, 2012. ^b^Source: Compost quality standards, Brinton W, 2000. ^c^Source: EU standards for arsenic, cadmium, lead, nitrites, volatile mustard oil and harmful botanical impurities, No 1275/2013, EU, 2013. ^d^Source: Standard for feeds, GB 13078–2001, 2001 and standard for copper as well as selenium (GB 26418–2010) in feeds, 2011. ^e^Source: Standard for feeds, Canadian Food Inspection Agency, 2015. ^f^Source: Standard for foodstuffs (No 1881/2006 and No 629/2008), EU. ^g^Source: Standard for contaminants and toxins in the GSCTFF, FAO/WHO, 2011. ^h^Source: Standard for contaminants in foodstuffs (GB 2762–2012), 2012. ^i^Heavy metal limit in meat/liver.

Heavy metal limits	Zn	Cu	Cr	Ni	As	Se	Pb	Cd
In fertilizer								
China^a^	—	—	150	—	15	—	50	3
Canada^b^	500	100	210	62	13	2	150	3
In animal feed								
European Union (EU)^c^	—	—	—	—	2	—	5	0.5
China^d^	—	35	10	—	2	0.5	5	0.5
Canada^e^	—	—	—	—	8	—	8	0.4
In human food								
European Union^f^	—	—	—	—	—	—	0.1/0.5^i^	0.05/0.5
WHO/FAO^g^	—	—	—	—	—	—	0.1/0.5	—
China^h^	—	—	1	—	0.5	—	0.2/0.5	0.1/0.5

**Table 3:** Heavy metal limits in fertilizer established by some European Union countries (mg/kg). ‘—’ means the standard of relevant heavy metal concentrations have not been stipulated.

	Zn	Cu	Cr	Ni	As	Pb	Se	Cd
Austria	1000	400	150	100	—	500	—	4
Belgium	1000	100	150	50	—	600	—	5
Denmark	—	—	—	45	25	120	—	1.2
France	—	—	—	200	—	800	—	8
Germany	400	100	100	50	—	150	—	1.5
Italy	500	300	100	50	10	140	—	1.5
Spain	4000	1750	750	400	—	1200	—	40

In the current study, the contents of the heavy metals in the maggots decreased rapidly with larval development, especially from 4 days old to 5 days old, except for that of Se (Fig. 2). Previous studies found that there are various metabolic immune responses in insects responsible for the detoxification of heavy metals. When *Spodoptera litura* were exposed to a range of Zn concentrations, the expression of the *metallothionein* (*MT*) gene was significantly positively correlated with the accumulation of Zn in the midgut^47^. Filipiak *et al*.^48^ found that a high concentration of Zn in the cytoplasm of *Drosophila melanogaster* haemocytes induces programmed cell death via a mitochondrial pathway and that caspases play a pivotal role in this process^48^. Wu and Yi (2015) analysed the effects of Cr and Pb on immune and antioxidant systems in *Galleria mellonella* and found that the activity of antioxidant enzymes (superoxide dismutase, peroxidase and catalase) significantly increased with increasing concentrations of dietary Cr and Pb^49^. Consequently, the BAF of all of the heavy metals decreased as the maggots grew. Distinct from the other heavy metals, the reductions in the BAF of Cd and Se were not obvious, and no Cr and Ni bioaccumulation was observed in 5-day-old maggots (Table 1). Similar results have been found regarding chickens fed with black soldier flies, *Hermetia illucens*, with the feed containing low concentrations of Cd, Pb, and Zn. The decrease in the BAF of Cd with the growth of the larvae was not significant in contrast to Pb and Zn^38^. Outcomes of our work indicated that the ability of maggots to accumulate heavy metals decreased with their growth. The heavy metal concentrations in insects were related to their growth stage and heavy metals previously absorbed could still be discharged from the bodies of maggots.

Comparing the concentrations of heavy metals in the harvested maggots used in the chicken feed in this study to the standards for animal feed in China, Canada, and the European Union (Table 2), the contents of Cu, Cr, As, Cd and Pb in the maggots met the standards in China, while the contents of Se and Cd exceeded the standards in China (Se < 0.5 mg/kg)^50–54^. The results indicated that the levels of heavy metals among the harvested maggots were distinctly lower than in the living environment, and regarding these five metals, these maggots would be safe to be used as feed.

Due to the contents in the chicken faeces (Fig. 3), we can conclude that the metabolic capacities of different heavy metals in chickens are diverse. Our results indicated that chickens have better excretion capacity for Cd, Se, Cu and Pb, with higher excretions in faeces with greater contaminant intake. On the other hand, the ability of chickens to excrete Ni, Cr, Zn and As is insufficient. Almost constant amount of Zn and As excretion or reduced of Ni and Cr excretion were calculated in our experiment meaning that the metabolic system might have been destroyed. Fortunately, overall concentrates of heavy metals in the faeces of chickens fed with 10% maggots, met the standard limits of fertilizer of Canada^45^, but the results remind us to strictly control the contents of Zn, As, Ni and Cu in chicken feed.

The liver is an important metabolic organ that is involved in detoxification. In our study, we found that the contents of Zn, Cu, Cd, and Se in the livers of chicken fed with feed containing 15% maggots were significantly higher than those in the meat (chest muscle), but for Cr, Ni, As and Pb, the concentrations were not significantly different (Fig. 4). We surmised that the livers contained more efficient mechanisms to metabolize Zn, Cu and Se since they are essential elements for life, and Cd might be processed in a similar way due to its similar location in the periodic table compared with Zn. For the other four elements (Pb, Cr, As and Ni), they don’t have similar properties. The contents of the heavy metals, Cr, As, Pb, and Cd, in the meat of chickens fed with 15% maggot meal harvested from swine manure, were 0.525, 0.003, 0.031, and 0.003 mg/kg, respectively, and those in the liver were 0.345, 0.02, 0.038, and 0.019 mg/kg. All the levels of the detected heavy metals met the standard limits of China, the European Union, and FAO/WHO^52–58^.

We concluded that insects can facilitate a decrease in the contents of some heavy metals in manure and that all the levels of heavy metals in maggot-treated manure met the standard limits in fertilizer. In addition, the harvested mature larvae are safe to be used as animal feed and provide abundant nutrients for their high protein content. The heavy metal species and their concentrations in insects depend on the manure substrate and the insect growth stage, which may subsequently affect higher trophic levels.

## Methods

### Conversion of swine manure by maggots

Manure was collected from a pig breeding farm at the Huazhong Agricultural University (30°29′N, 114°22′E). *Musca domestica* flies were incubated at 26 ± 2 °C and 60% relative humidity (RH) in a cubic cage (60 cm × 50 cm × 40 cm) with a photoperiod of 13:11 (L:D) h. Sugar, water, and milk powder were provided starting from first adult emergence. The eggs that were laid by the adult flies were collected along with the swine manure and wrapped with cloth, and one gram of eggs was moved into a breeding box (65 cm × 40 cm × 15 cm) with four kilograms of swine manure. Five-day-old maggots were harvested, starved for 12 h, weighed, and dried at 60 °C for the chicken feeding experiment, while maggots at 3, 4, and 5 days of age, raw manure, and maggot-treated manure were sampled for heavy metal analysis, with each experiment being replicated 3 times.

### Chicken trial

One-day-old chickens were purchased from the hatcheries of the Guangdong Wen Group, located in Jiangxia, Wuhan (29°58′N, 113°42′E). Maggot meal was added to the chicken feed as an alternative protein source at 0%, 5%, 10%, and 15% of the total protein. Seven-day-old chickens with an average weight of 58.26 ± 0.17 g were starved for 4 h to empty their digestive tracts. Eighty-four chickens were randomly allocated to the 4 dietary treatments, which were replicated 3 times, to study the effect of including maggots harvested from swine manure in the diet on the heavy metal content of chickens and chicken manure. Chicken manure was collected daily and combined for heavy metal analysis, and after seven weeks, the meat (chest muscle) and livers of the chicken fed with 15% maggot meal were sampled for heavy metal analysis.

### Ethics statement

All methods were carried out in accordance with the guidelines approved by Huazhong Agricultural University. All animal experiments were performed under the guidelines of the Committee on the Ethics of Animal Experiments of Biological Studies Animal Care and Use Committee in Hubei Province.

### Metal analysis

The samples (maggots at 3, 4, and 5 days of age starved for 8 h, and the meat and livers of chickens at 43 days of age) were washed with deionised water, weighed, dried to measure the dry weight, and then ground in an agate mortar for heavy metal analysis. The manure samples (raw swine manure, maggot-treated swine manure, manure from chickens fed with maggot meal) were treated in the same manner except for the washing. Sample pre-treatment was conducted following the method of Diener *et al*. (2015)^19^. Samples were analysed through an inductively coupled plasma mass spectrometry (ICP-MS, Agilent 7700 series, USA).

### Statistics

Statistical analyses were performed using SPSS Statistics 19.0 software (SPSS Inc., Chicago, IL, USA). Student’s *t*-test was used to analyse the significant differences in the heavy metal content between the raw swine manure and the maggot-treated manure and between the meat and livers of the chickens fed 15% maggot meal. *ANOVA* was used to detect statistically significant differences in the heavy metal content among the maggots of different ages. The BAF was calculated according to Walker (1990) as follows:

1 $${\rm{BAF}}=\frac{{\rm{Concentration}}\,{\rm{in}}\,{\rm{organism}}({{\rm{C}}}_{{\rm{i}}})}{{\rm{Concentration}}\,{\rm{in}}\,{\rm{ingested}}\,{\rm{food}}\,{\rm{and}}/{\rm{or}}\,{\rm{water}}\,({{\rm{C}}}_{{\rm{0}}})}$$

In this research, C_0_ represented the concentration of heavy metals in the swine manure, and C_i_ represented the concentration of heavy metals in the harvested larvae.
